# Efficient Incorporation of Unnatural Amino Acids into Proteins with a Robust Cell-Free System

**DOI:** 10.3390/mps2010016

**Published:** 2019-02-12

**Authors:** Wei Gao, Ning Bu, Yuan Lu

**Affiliations:** 1 College of Life Science, Shenyang Normal University, Shenyang 100034, Liaoning, China; weigao1122@gmail.com; 2 Department of Chemical Engineering, Tsinghua University, Beijing 100084, China; 3 Institute of Biochemical Engineering, Department of Chemical Engineering, Tsinghua University, Beijing 100084, China; 4 Key Lab of Industrial Biocatalysis, Ministry of Education, Department of Chemical Engineering, Tsinghua University, Beijing 100084, China

**Keywords:** cell-free protein synthesis, unnatural amino acid, unnatural protein

## Abstract

Unnatural proteins are crucial biomacromolecules and have been widely applied in fundamental science, novel biopolymer materials, enzymes, and therapeutics. Cell-free protein synthesis (CFPS) system can serve as a robust platform to synthesize unnatural proteins by highly effective site-specific incorporation of unnatural amino acids (UNAAs), without the limitations of cell membrane permeability and the toxicity of unnatural components. Here, we describe a quick and simple method to synthesize unnatural proteins in CFPS system based on *Escherichia coli* crude extract, with unnatural orthogonal aminoacyl-tRNA synthetase and suppressor tRNA evolved from *Methanocaldococcus jannaschii*. The superfolder green fluorescent protein (sfGFP) and *p*-propargyloxyphenylalanine (*p*PaF) were used as the model protein and UNAA. The synthesis of unnatural sfGFPs was characterized by microplate spectrophotometer, affinity chromatography, and liquid chromatography-mass spectrometry/mass spectrometry (LC-MS/MS). This protocol provides a detailed procedure guiding how to use the powerful CFPS system to synthesize unnatural proteins on demand.

## 1. Introduction

Protein is a vital class of biomolecules, and all living organisms employ it to fulfill essential structural, functional, and enzymatic roles to sustain life [1]. In nature, 20 natural amino acids can form proteins in a near-infinite number of combinations to make them have structural and functional diversity [1]. However, these natural amino acids can possess some interesting chemistries [2,3]. Thus, expanding protein functions by incorporating unnatural amino acids (UNAAs) featuring novel functional groups is more important [4].

Methods of UNAA incorporation usually comprise global suppression, amber suppression, frame-shift suppression, sense codon reassignment, and unnatural base-pairs; the most popular approach is amber suppression [5]. Usually, unnatural orthogonal translation systems (OTS) are designed to incorporate UNAAs in vivo or in vitro [6,7]. OTS, is an orthogonal system which can covalently load UNAA onto the suppressor tRNA (o-tRNA) via acylation of aminoacyl-tRNA synthetase (o-aaRS) [1]. To date, many various UNAAs have been incorporated into proteins successfully by amber suppression with OTSs [1,8]. Thus, expanding the genetic code by incorporating UNAAs has become a significant opportunity in synthetic and chemical biology [3]. In addition, unnatural proteins have been applied in protein modifications [9], biophysical probes [10], enzyme engineering [11], biomaterials [12] and biopharmaceutical protein production [13]. The synthesis of unnatural proteins can be based on two systems. One is an in vivo cellular system, and the other is an in vitro cell-free protein synthesis (CFPS) system. In conventional in vivo systems, incorporation of UNAAs is dependent on cell viability, and the cell membrane barrier limits the transportation of UNAAs [14]. Compared with in vivo system, a CFPS system has several apparent advantages [7]. A CFPS system consists of crude extract with basal transcription and translation elements, amino acids, DNA templates, energy regeneration substrates, nucleotides, salts, and cofactors [15]. The transcription and translation processes happen in an open biological reaction system. Because there is no limitation of the cell membrane and no cell growth, the CFPS system has higher utilization efficiency of UNAAs, higher flexibility of reactions, higher toxicity tolerance, shorter production cycle [16], and higher protein yield [17]. Therefore, we can combine the CFPS system with the OTS system to highly effective produce unnatural proteins with UNAA by amber suppression.

## 2. Experimental Design

**Figure 1:**
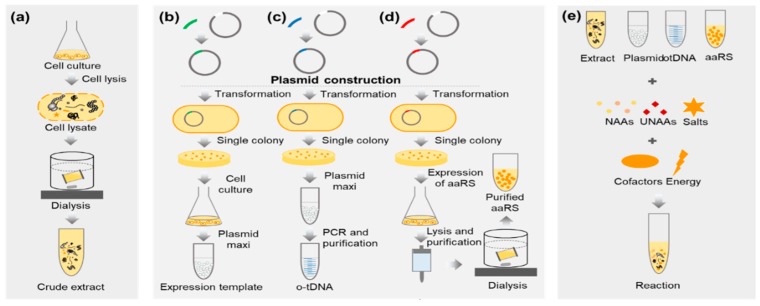
Experimental design. (**a**) Preparation of crude extract; (**b**) extraction of expression templates; (**c**) purification of o-tDNA; (**d**) purification of aaRS; (**e**) cell-free protein synthesis reaction.

This protocol describes how to synthesize unnatural proteins with UNAAs. This method is based on a robust CFPS platform, and combines the expansion of the genetic code with unnatural OTSs to achieve the incorporation of UNAAs. In this study, the superfolder green fluorescent protein (sfGFP) was used as the model protein, in which position 2 was chosen for the UNAA incorporation. In this protocol, the incorporation of UNAAs can be preliminarily determined via the fluorescence intensity of unnatural sfGFP; thus, the selected position must not destroy the chromophore. Additionally, a previous study indicated that the yield of unnatural protein that contains UNAA at position 2 is approximate to the yield of the wild-type protein [11]. The experiment is carried out in four steps (See Figure 1). First, crude cell lysate was made. Second, OTS including aminoacyl-tRNA synthetase of *p*-propargyloxyphenylalanine (*p*PaFRS) and o-tDNA^opt^ was made. Third, CFPS reaction components were formulated. Lastly, unnatural proteins were synthesized and characterized. The following section describes the specific experimental procedures.

### 2.1. Materials

#### 2.1.1. Preparation of *E. coli* Extract

- *E. coli* Rosetta (DE3) strain (Biomed, Beijing, China; Cat. no.: BC204-01)
- Antifoam 204 (Sigma-Aldrich, St. Louis, MO, USA; Cat. no.: SLBW1473)
- Chloramphenicol (Genview, Beijing, China; Cat. no.: AC060)
- Tryptone (OXIOD, Basingstoke, UK; Cat. no.: LP0042)
- Yeast extract (OXIOD; Cat. no.: LP0021)
- Bacto^TM^ agar (Becton. Dickinson, Franklin Lakes, NJ, USA; Cat. no.: 7291815)
- NaCl (Sinopharm Chemical Reagent, Shanghai, China; Cat. no.: 10019318)
- Dipotassium hydrogen phosphate (KH_2_PO_4_) (Tong Guang Fine Chemicals, Beijing, China; Cat. no.: 7778-77-0)
- Potassium hydrogen phosphate trihydrate (K_2_HPO_4_·3H_2_O) (Tong Guang Fine Chemicals; Cat. no.: 16788-57-1)
- Ampicillin (Solarbio, Beijing, China; Cat. no.: A8180)
- Potassium L-glutamate (Yuanye, Shanghai, China; Cat. no.: S20427)
- L-Glutamic acid hemimagnesium salt tetrahydrate (Sigma-Aldrich; Cat. no.: 49605)
- Tris (Biotopped, Beijing, China; Cat. no.: T6061)
- 1,4-Dithio-DL-threitol (DTT) (Solarbio; Cat. no.: D8220)

#### 2.1.2. Preparation of *p*PaFRS

- The nucleotide sequence of *p*PaFRS refers to pPRMjRS-1 [18]
- Plasmid pEVOL-pAzF [19] (pEVOL-pAzF was a gift from Peter Schultz (Addgene plasmid #31186; http://n2t.net/addgene:31186; RRID: Addgene_31186))
- Plasmid pET24a (Novagen, Shanghai, China; Cat. no.: 69749-3)
- Primers P1f, P1r, P2f, P2r, P3f and P3r (See Table S1)
- Q5^®^ High-Fidelity DNA Polymerases (New England Biolabs, Beijing, China; Cat. no.: M0491)
- *E. coli* strain expressing the *p*PaFRS gene: BL21(DE3) competent cells (Biomed; Cat. no.: BC201)
- Kanamycin (Solarbio; Cat. no.: K8020)
- Tryptone (OXIOD; Cat. no.: LP0042)
- Yeast extract (OXIOD; Cat. no.: LP0021)
- BactoTM agar (Becton. Dickinson; Cat. no.: 7291815)
- NaCl (Sinopharm Chemical Reagent; Cat. no.: 10019318)
- Isopropyl-b-D-thiogalactoside (Solarbio; Cat. no.: I8070)
- EzFast Ni HP) columns (5 mL) (BestChrom, Shanghai, China; Cat. no.: EA005)
- Ethanol (TONG GUANG FINE CHEMICALS; Cat. no.: 32061)
- Imidazole (Sigma-Aldrich; Cat. no.: I2399)
- Quick Start Bradford Protein Assay Kit (Bio-Rad, Hercules, CA, USA; Cat. No.: 5000201)
- PBS buffer (Solarbio; Cat. no.: P1010)

#### 2.1.3. Preparation of Crude T7 RNA Polymerase

- Plasmid pAR1219 (Sigma-Aldrich; Cat. no.: T2076).
- *E. coli* strain expressing the T7 RNA polymerase gene: BL21(DE3) competent cells (Biomed; Cat. no.: BC201)
- Tryptone (OXIOD; Cat. no.: LP0042)
- Yeast extract (OXIOD; Cat. no.: LP0021)
- BactoTM agar (Becton. Dickinson; Cat. no.: 7291815)
- NaCl (Sinopharm Chemical Reagent; Cat. no.: 10019318)
- Potassium acetate (Sigma-Aldrich; Cat. no.: V900213)
- Magnesium acetate tetrahydrate (Sigma-Aldrich; Cat. no.: V900172)
- Ethylenediaminetetraacetic acid (EDTA) (Solarbio; Cat. no.: E8040)
- 1,4-Dithio-DL-threitol (DTT) (Solarbio; Cat. no.: D8220)
- Potassium hydrogen phosphate trihydrate (K_2_HPO_4_·3H_2_O) (Tong Guang Fine Chemicals; Cat. no.: 16788-57-1)
- β-mercaptoethanol (Amresco, Shanghai, China; Cat. no.: 0482)
- 1 × Protease inhibitor (Sigma-Aldrich; Cat. no.: P8340)

#### 2.1.4. Preparation of Expression Template and o-tDNA^opt^

- Plasmid pET23a (Novagen; Cat. no.: 69745-3)
- Primers P4f and P4r (See Table S1) to generate pET23a-sfGFP-StrepII gene.
- Primers P5f and P5r (See Table S1) to generate pET23a-sfGFP(2TAG)-StrepII gene which contained the TAG site in the 2nd codon and C-terminal StrepII tag.
- QIAGEN Plasmid Maxi Kit (10) (QIAGEN, Shanghai, China; Cat. no.: 12162)
- Plasmid Mini Kit (OMEGA Bio-Tek, Atlanta, GA, USA)
- The o-tDNA^opt^ gene (GENEWIZ, Suzhou, China)
- Primers P6f and P6r to amplify the pET23a vector gene (See Table S1)
- Primers P7f and P7r to amplify the single o-tDNA^opt^ gene [20] which can ligate with pET23a vector gene (See Table S1)
- Pfu polymerase (Beyotime Biotechnology, Shanghai, China; Cat. no.: D7217)
- Ethanol (Tong Guang Fine Chemicals; Cat. no.: 32061)
- 3 M Sodium acetate (Solarbio; Cat. no.: A1070)

#### 2.1.5. Synthesis and Characterization of sfGFP and sfGFP2*p*PaF

All the chemicals were from Sigma except special description.

- 10 × salt: 1.75 M Potassium glutamate, 27 mM Potassium oxalate monohydrate, 100 mM Glutamate, adjusted the pH to 7.2~7.4 with ammonia while dissolving
- Mg^2+^ solution: 1 M Magnesium glutamate.
- Amino acids mixture: Added the amino acids in the following order (given in three-letter code): Arg, Val, Trp, Phe, Ile, Leu, Cys, Met, Ala, Asn, Asp, Gly, Gln, His, Lys, Pro, Ser, Thr, Tyr. During preparation, it was necessary to ensure that any of amino acids has been dissolved before adding the next, and Tyr was added at last. Adjusted the pH to 7.4 with ammonia hydroxide.
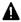
**CRITICAL STEP** Phosphoenolpyruvic acid (PEP) solution: (Prepare rapidly on the ice and flash frozen) 883 mM Phosphoenolpyruvate, added 10 M KOH to adjust pH to 7.4.
- GSSG (Oxidized glutathione) and GSH (Reduced glutathione): 100 mM GSSG, 100 mM GSH.
- 25 × NTPs mixture: Prepared and added all reagents one by one as the following order: 1 M putrescine, 1.5 M spermidine, 8.3 mM NAD, 30 mM ATP, 21.5 mM CTP, GTP and UTP, 6.8 mM CoA, 4.3 mg/mL *E. coli* tRNA, and 0.9 mg/mL folinic acid. Before adding the next reagent, ensure the last reagent was completely dissolved. The final pH should be between 7.4 and 7.6. Store at −80 °C.
- *p*PaF solution: 100 mM (Medchem Source LLP, Washington, America; Cat. no.: JA-1003).
- *E. coli* extract (Prepared in 3.1).
- The *p*PaFRS (Prepared in 3.2).
- 25 mM of DTT.
- 55 mM iodoacetamide.
- 0.1% trifluoroacetic acid in 50% acetonitrile aqueous solution.
- Sequencing grade modified trypsin (Promega, America; Cat. No. #V5117).
- Mobile phase A: 0.1% formic acid.
- Mobile phase B: 100% acetonitrile and 0.1% formic acid.

### 2.2. Equipment

#### 2.2.1. Preparation of *E. coli* Extract

- 1 L flasks.
- Constant temperature shaker.
- BIOSTAT^®^ A plus Bioreactor (Sartorius, Gottingen, Germany)
- Ultrospec 3100 pro UV/Visible spectrophotometer (Amersham, Piscataway, NJ, USA)
- Micro Refrigerated Centrifuge Model 3700 (KUBOTA, Osaka, Japan)
- Vortex-Genie 2 (Scientific Industries, Bohemia, NY, USA)
- JN-3000PLUS high press crusher (JNBIO, China)
- Spectra/Por #1 dialysis tubing, MWCO 6-8 kD (Spectrum Laboratories, Rancho Dominguez, CA, USA)
- MS-H-Pro+ magnetic stirring apparatus (DRAGONLAB, Beijing, China)

#### 2.2.2. Preparation of *p*PaFRS

- 1 L flasks.
- Constant temperature shaker.
- JN-3000PLUS high press crusher (JNBIO)
- Ultrospec 3100 pro UV/Visible spectrophotometer (Amersham)
- Micro Refrigerated Centrifuge Model 3700 (KUBOTA)
- Vortex-Genie 2 (Scientific Industries)
- ÄKTAprime plus (GE Healthcare, Chicago, IL, USA)
- MS-H-Pro+ magnetic stirring apparatus (DRAGONLAB)
- Amicon Ultra 15 mL 10 K (Merck Millipore, Darmstadt, Germany; Cat. No. UFC901096)
- Orbital Shaker TS-2 (Kylin-Bell Lab Instruments, Haimen, China)

#### 2.2.3. Preparation of Crude T7 RNA Polymerase

- 1 L flasks.
- Constant temperature shaker.
- Ultrospec 3100 pro UV/Visible spectrophotometer (Amersham)
- Micro Refrigerated Centrifuge Model 3700 (KUBOTA)
- Vortex-Genie 2 (Scientific Industries)
- Qsonica Q700 Ultrasonic crusher (Misonix, Farmingdale, NY, USA)
- MS-H-Pro+ magnetic stirring apparatus (DRAGONLAB)

#### 2.2.4. Preparation of Expression Template and o-tDNA^opt^

- Constant temperature shaker.
- Micro Refrigerated Centrifuge Model 3700 (KUBOTA)
- Vortex-Genie 2 (Scientific Industries)
- Plasmid mini kit (Omega Bio-Tek)
- C100TM Thermal Cycler (Bio-Rad)
- NanoVue Plus (GE Healthcare)

#### 2.2.5. Synthesis and Characterization of sfGFP and sfGFP2*p*PaF

- Infinite M200 PRO Microplate reader (Tecan, Switzerland)
- Strep-Tactin affinity chromatography (IBA GmbH, Goettingen, Germany)
- Amicon Ultra 15 mL 10 K (Merck Millipore; Cat. No. UFC901096)
- Mini-PROTEAN^®^ Tetra Cell (Bio-rad)
- SpeedVac (Thermo Fisher Scientific, Waltham, MA, USA)
- EASY-nLC 1000 system (Thermo Fisher Scientific)
- Analytical column: A home-made fused silica capillary column (75 µm ID, 150 mm length; Upchurch, Oak Harbor, WA, USA) packed with C-18 resin (300 Å, 5 µm, Varian, Lexington, MA)
- Orbitrap Fusion Tribrid mass spectrometer (Thermo Fisher Scientific, Bremen, Germany)
- Xcalibur3.0 software
- Proteome Discoverer (Version PD1.4, Thermo Fisher Scientific)

## 3. Procedure

### 3.1. Preparation of E. coli Extract (Time for Completion: 2 Days)

The volume of medium is one-fifth of the volume of flasks.

1. Prepared solutions used in this part.
  (1) 2 × Yeast extract-Tryptone (YT)-Phosphate (P)-Chloramphenicol (Cm) medium (10 g/L Yeast extract, 16 g/L Tryptone, 5 g/L NaCl, 40 mM K_2_HPO_4_, 22 mM KH_2_PO_4_, 34 mg/L chloramphenicol, and 1.5% agar for plate use if needed).
  (2) S30 buffer A: 14 mM L-Glutamic acid hemimagnesium salt tetrahydrate, 60 mM Potassium L-glutamate, 50 mM Tris, pH 7.7, titrated with acetic acid. Add DTT to 2 mM just before use. Stored at 4 °C.
  (3) S30 buffer B: 14 mM L-Glutamic acid hemimagnesium salt tetrahydrate, 60 mM Potassium L-glutamate, pH 8.2, titrated with Tris. Add DTT to 1 mM just before use. Stored at 4 °C.
2. Prepared and autoclaved 2 × YT-P medium (10 mL, 200 mL, and 4 L in the bioreactor) and S30 A buffer (500 mL) and S30 B buffer (2 L).
3. Cultivated *E. coli* Rosetta(DE3) strain on 2 × YT-P-Cm solid medium, and incubated overnight at 37 °C.
4. Selected single colony and transferred it to 10 mL liquid 2xYT-P medium in 50 mL flask with Cm and incubated overnight at 37 °C with 220 rpm.
5. Transferred 10 mL overnight culture into 200 mL fresh 2xYT-P medium in 1 L flask and continued culturing for about 2 h in the shaker.
6. When the OD600 of culture reached to 2 to 3, it was transferred into a 4-L bioreactor (Sartorius) with Cm and 400 μL antifoam. Controlled the fermentation conditions at 37 °C and 500 rpm stirring.
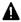
**CRITICAL STEP** When the OD600 reached to 3.5 to 4.0, harvested the cells quickly at 4 °C to obtain high activity of cell extracts.
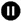
**PAUSE STEP** Washed the cell pellets with 100 mL S30 buffer A at least twice (after being washed, the pellet could be stored at 4 °C overnight or at −80 °C for a long time).
9. Re-suspended the pellets in 1 mL of S30 buffer A per gram of biomass on the ice.
10. Subjected the suspension to a high press crusher (JNBIO) twice at 15000~20000 psi.
11. Centrifuged the lysed cells at 4 °C and 13,000× *g* for at least 30 min.
12. Incubated the supernatant at 37 °C with 120 rpm for 80 min.
13. Centrifuged the extract at 4 °C and 13,000× *g* for at least 30 min.
14. Transferred the supernatant to 6–8 kDa MWCO dialysis tubing and did dialysis in 100 times volume of supernatant S30 buffer B overnight at 4 °C (or 4 h twice).
15. Re-centrifuged the extract at 4 °C with 13,000× *g* for 30 min.
16. Collected and transferred the supernatant to 1.5 mL Eppendorf tubes on ice.
17. Flash-frozen the extracts in liquid nitrogen and stored them at −80 °C.

### 3.2. Preparation of pPaFRS (Time for Completion: 5~6 Days from Plasmid Construction to pPaFRS Purification)

The volume of the medium is one-fifth of the volume of flasks.

1. Prepared buffers used in this part.
  (1) His-tag binding buffer: 30 mM Imidazole, 20 mM Na_3_PO_4_, 500 mM NaCl, titrated with phosphoric acid to pH 7.4. Stored at 4 °C.
  (2) His-tag elution buffer: 500 mM Imidazole, 20 mM Na_3_PO_4_, 500 mM NaCl, titrated with phosphoric acid to pH 7.4. Stored at 4 °C.
2. Amplified the pET24a vector and *p*PaFRS gene by Q5^®^ High-Fidelity DNA Polymerases system.
3. Ligated the pET24a vector and *p*PaFRS gene with the homologous arm at 50 °C and 15 min.
4. Screened with LB-K plate (LB plate with 50 μg/mL Kanamycin) by DH5α.
5. Selected a single colony and sequenced it.
6. Transformed pET24a-6H-pPaFRS into *E. coli* BL21(DE3), spread the cell on a LB-K plate, and incubated overnight at 37 °C.
7. Picked up a single colony with a toothpick and inoculated it directly into 10 mL of LB-K medium followed by incubation for 12 h at 37 °C and 220 rpm.
8. Transferred 10 mL culture into 200 mL LB-K medium and continued culturing it overnight in a shaker.
9. Transferred the cells into 1 L fresh LB-K medium at a 5% inoculation amount.
10. When the OD600 reached 0.6–0.8, added 1 mM of Isopropyl β-D-Thiogalactoside (IPTG).
11. The cells were further cultured for 3–4 h at 37 °C. Cultivated the cells at 4 °C and 10,000 × *g*.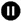 PAUSE STEP Washed the cells with 100 mL His-tag binding buffer at least twice (after being washed, the pellets could be stored at 4 °C overnight or at −80 °C for a long time).
13. Re-suspended the pellets with suitable His-tag binding buffer, making the final optical density at a wavelength of 600 nm (OD600) between 40 and 60 on ice.
14. Subjected the suspension to high press crusher single time at 15,000~20,000 psi.
15. Centrifuged the lysate at 4 °C and 13,000 × *g* for 30 min.
16. Filtrated the lysate with 0.45 μm water filters.
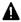
**CRITICAL STEP** All the lysate was loaded onto a 5 mL EzFast Ni HP column, which was connected with the ÄKTA Prime system and equilibrated with His-tag binding buffer. Then, the target *p*PaFRS was eluted with His-tag elution buffer and collected eluate in 1 mL fractions (all buffers should eliminate bubbles and be stored at 4 °C).
18. Placed the eluate in the 6–8 kDa MWCO dialysis tubing and dialyzed it against 50–100 volumes of sterile PBS (pH 7.4) buffer overnight.
19. Determined protein concentrations using Quick Start Bradford Protein Assay Kit. When necessary, the fractions were concentrated using Amicon Ultra centrifugal device (10 kDa).
20. Added 20% (*v/v*%) sucrose to fractions, and stored at −80 °C.

### 3.3. Preparation of Crude T7 RNA Polymerase (Time for completion: 3 days)

The volume of medium is one fifth of the volume of the flasks.

1. Prepared buffers used in this part (stored at 4 °C).
  (1) Lysis buffer: 50 mM NaCl, 10 mM EDTA, 10 mM K_2_HPO_4_, 1 mM DTT, 10 mM β- mercaptoethanol, 1 × Protease inhibitor, 5% glycerin, pH 8.0.
  (2) Dialysis buffer: 50 mM NaCl, 1 mM EDTA, 40 mM K_2_HPO_4_, 1 mM DTT, 20% Sucrose, pH 7.7.
2. Transformed *E. coli* BL21(DE3) with pAR1219, and incubated it on LB plate with 100 μg/mL Ampicillin (LB-A) overnight at 37 °C.
3. Selected a single colony to inoculate in 10 mL of LB-A medium and cultured cells for 12 h at 37 °C and 220 rpm.
4. Transferred 10 mL culture into 100 mL LB-A medium and continued culturing cells overnight in a shaker.
5. Transferred cells into fresh LB-A medium at a 5% inoculation amount.
6. When OD600 reached 0.6–0.8, IPTG was added to a final concentration of 0.1 mM.
7. When OD600 reached 2.0, cells were harvested for 10,000 × *g* at 4 °C.
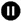
**PAUSE STEP** The cells were washed with 5 mL ice-cold wash buffer per gram pellet for twice (after washing, the pellet can be stored at 4 °C overnight or at −80 °C for a long time).
9. Re-suspended in 4 mL ice-cold lysis buffer per gram cells.
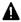
**CRITICAL STEP** Cells were ultrasonicated for 40 min on the ice working for two seconds and intermittent for two seconds (Kept the sample on the ice to maintain the activity).
11. Cells were centrifuged at 13,000 × *g* and 4 °C for 20 min and discarded cell pellet.
12. Dialyzed (6–8 kDa) twice in the dialysis buffer (100 times volume of samples) at 4 °C overnight. The suspension was centrifuged at 10,000× *g* for 30 min at 4 °C and the pellet was discarded.
13. The crude T7 RNA polymerase was flash-frozen in liquid nitrogen, and stored at −80 °C until use.

### 3.4. Preparation of Expression Template and o-tDNA^opt^ (Time for Completion: 2 Days)

1. Made pET23a-sfGFP-StrepII and pET23a-sfGFP(2TAG)-StrepII plasmids self-ligated by homology arms at 50 °C at least 15 min.
2. Added the ligation system into 100 μL DH5α competent cells and put them on ice for 30 min.
3. Added LB medium about 400 μL and cultured them at 37 °C and 220 rpm about 30 min.
4. Coated about 100 μL suspension on the LB-A plate.
5. Picked up two single colonies to sequence.
6. Selected the correct strain and extracted the plasmid with QIAGEN Plasmid Maxi Kit.
7. Ligated the o-tDNA^opt^ and the pET23a vector genes at 50 °C at least 15 min.
8. Screening method was the same as 3.4 2–5.
9. Extracted the pET23a o-tDNA^opt^ with Plasmid Mini Kit as an o-tDNA^opt^ amplification template.
10. Amplified o-tDNA gene with Pfu polymerase.
11. Purified o-tDNA gene by ethanol precipitation.
12. The o-tDNA^opt^ was diluted with MiliQ water to 2 mg/mL and stored at –20 °C.

### 3.5. Synthesis and Characterization of sfGFP and sfGFP2pPaF (Time for Completion: 3~4 Days)

1. Thawed the CFPS, OTS components, and expression templates on the ice.
2. The standard 20 μL cell-free reaction mixture consisted of the followings in Table 1.
3. Mixed the mixture and reacted at 30 °C for 16 h.
4. Five-microliter samples from each reaction mixture were diluted with 195 μL ddH_2_O, and the fluorescence intensity of these diluted samples was measured with F485 excitation and F535 emission filters using Microplate reader.
5. Preparation of samples for mass spectrometry:
  (1) Purified sfGFP and sfGFP2*p*PaF produced in CFPS with a C-terminal Strep-tag via Strep-Tactin affinity chromatography according to the manufacturer instructions.
  (2) Dialyzed and concentrated the fractions obtained from Strep-Tactin affinity chromatography with PBS (pH 7.4) buffer at 4 °C.
  (3) Determined protein concentrations using Quick Start Bradford Protein Assay Kit.
  (4) Analyzed the samples by sodium dodecyl sulfate polyacrylamide gel electrophoresis (SDS-PAGE).
  (5) Extracted interested bands from the gel.
  (6) Reduced with 25 mM of DTT and alkylated with 55 mM iodoacetamide and digested in gel with sequencing grade modified trypsin at 37 °C overnight.
  (7) Extracted peptides twice with 0.1% trifluoroacetic acid in 50% acetonitrile aqueous solution for 30 min and then dried in a SpeedVac.
  (8) Redissolved peptides in 25 μL 0.1% trifluoroacetic acid.
  (9) Analyzed 6 μL of extracted peptides by Thermo orbitrap fusion.
  (10) LC-MS/MS analysis of samples was performed at the Center of Biomedical Analysis of Tsinghua University.

## 4. Expected Results

### 4.1. Preparation of pPaFRS and o-tDNA^opt^

**Figure 2:**
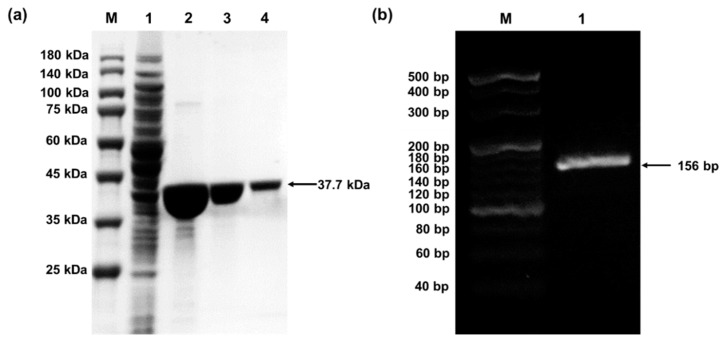
(**a**) SDS-PAGE result of *p*PaFRS. M: protein marker (PM2510, Transgen, China); 1: Cell lysate of induced BL21(DE3); 2, 3, and 4: different loading amounts of *p*PaFRS. (**b**) The o-tDNA gene by Polymerase Chain Reaction (PCR) amplification. M: 20 bp DNA Ladder (TaKaRa, Japan; Cat. No. #3420A). 1: The product of the o-tDNA gene by PCR.

Good preparation of *p*PaFRS and o-tDNA^opt^ is the first step to successful incorporation of UNAAs. *p*PaFRS proteins were purified by His-tag affinity chromatography, concentrated to 10 mg/mL by ultrafiltration tube, and stored with 20% sucrose. From the SDS-PAGE result (Figure 2a), the only protein band indicated successful purification. The o-tDNA genes were amplified by PCR and purified by ethanol precipitation. Analyzed by 1% agarose electrophoresis (Figure 2b), the o-tDNA gene was the only product. Thus, ethanol precipitation used in this study was suitable for o-tDNA purification, and this product was favorable for subsequent CFPS reaction.

### 4.2. Synthesis and Characterization of sfGFP and sfGFP2pPaF

#### 4.2.1. Synthesis and Characterization of sfGFP and sfGFP2*p*PaF

**Figure 3:**
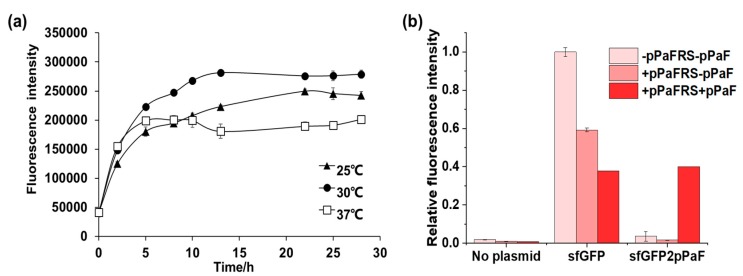
(**a**) Time course of sfGFP2*p*PaF synthesis catalyzed by purified OTS. Two independent batch CFPS reactions (n = 2) were performed at 25 °C, 30 °C and 37 °C for each point over 24 h. (**b**). Synthesis of sfGFP2*p*PaF at 30 °C for 16 h.

To screen the optimum conditions, reactions were conducted at 25 °C, 30 °C, and 37 °C, and then, the fluorescence intensity was measured at different time points (Figure 3a). According to the results, the reaction proceeded fastest at 37 °C; however, the fluorescence intensity was the weakest among the three different temperatures. At 25 °C, the reaction kept steady after 22 h, and the fluorescence intensity was weaker than at 30 °C. The fluorescence intensity was the strongest at 30 °C and kept steady after 13 h. Therefore, the optimum reaction temperature was 30 °C.

To further investigate the incorporation efficiency, reactions were conducted at 30 °C in 16 h. The experiment was designed into three groups. The first group was without *p*PaFRS and *p*PaF. The second was only *p*PaFRS without *p*PaF. The last group was added with both *p*PaFRS and *p*PaF. When the pET23a-sfGFP(2TAG)-StrepII plasmid was added into cell-free reactions, the fluorescence of the last group with both *p*PaFRS and *p*PaF should increase obviously; however, the other two groups should present weaker fluorescence. As shown in Figure 3b, this indicated that the addition of OTS components could reduce the expression of sfGFP by 40% to 60%, which suggested that the unnatural OTS components indeed were harmful to the natural system. It could be observed that more rigorous incorporation has been achieved because the fluorescence intensity of sfGFP2*p*PaF experimental group was far stronger than its control group. In brief, *p*PaF could be efficiently incorporated by this protocol.

#### 4.2.2. Characterization of sfGFP and sfGFP2*p*PaF

**Figure 4:**
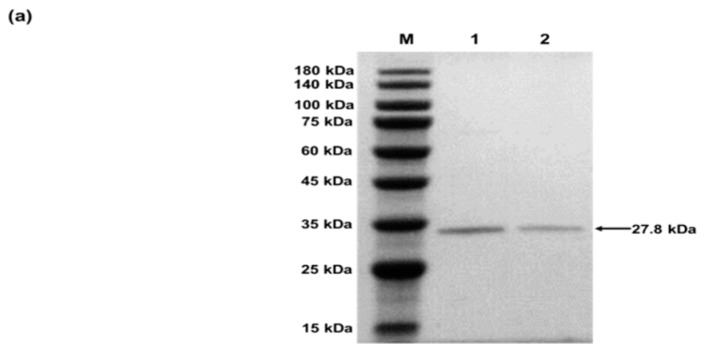

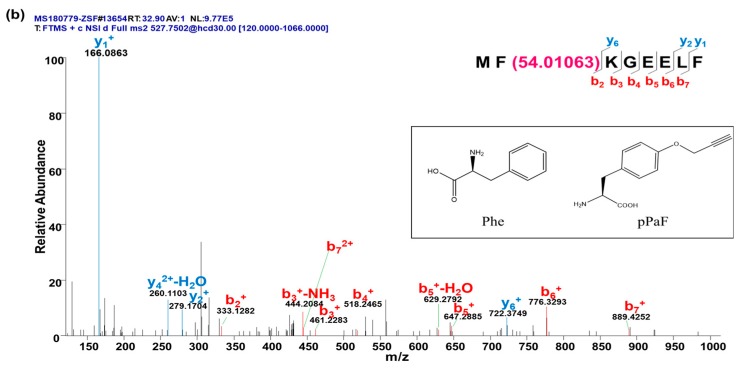
(**a**) Purified protein with C-terminal Strep-tag. 1: wild-type sfGFP; 2: sfGFP2*p*PaF. (**b**) LC-MS/MS analysis of sfGFP2*p*PaF.

The unnatural proteins produced in CFPS system need to be further characterized. Wild-type sfGFP and unnatural sfGFP2*p*PaF proteins were first purified by Strep-Tactin affinity chromatography. Fractions obtained from Strep-Tactin affinity chromatography were analyzed by SDS-PAGE and LC-MS/MS. The SDS-PAGE results (Figure 4a) revealed that the target protein has only one band, which meant successful protein purification. As shown in Figure 4b, mass spectrometry detected modification of an alkynyl group at the original second position and a molecular weight increase of 54.01063. The molecular weight of *p*PaF is 54.01063 higher than that of Phe. The LC-MS/MS analysis confirmed that *p*PaF had been successfully incorporated into the sfGFP protein.

## 5. Conclusions

This article described a method of synthesizing unnatural proteins that contained UNAAs at a specific single position. Compared with previous methods, this method used OTS components separately and achieved more controllable and efficient incorporation of UNAAs. Therefore, the whole experimental cycle could be shortened and need not prepare cell extracts expressing different OTSs. In this strategy, o-tRNA was added indirectly by o-tDNA transcription promoted by T7 promoter, which avoided redundant transcription in vitro. Consequently, this method is quick, convenient, and highly efficient, and it could be developed as the standard protocol for unnatural protein synthesis.

## Figures and Tables

**Table 1:** Components of standard cell-free protein synthesis (CFPS) system.

Components	Volume (μL)
10 × salt	2
PEP	1.6
Mg^2+^	0.4
Extract	5
T7 polymerase	0.2
NTPs mixture	0.8
Amino acids mixture	0.8
Plasmid template	1 (300 ng)
o-tDNA	1 (100 ng/μL)
*p*PaFRS	1 (5 mM)
ddH_2_O	6.12
Total	20

PEP: Phosphoenolpyruvic acid; pPaFRS: p-propargyloxyphenylalanine.

## Supplementary Materials

The following are available online at https://www.mdpi.com/2409-9279/2/1/16/s1, Figure S1: Nucleotide and amino acid sequences of *p*PaFRS, Figure S2: Nucleotide and amino acid sequences of original sfGFP, Figure S3: Nucleotide and amino acid sequences of mutational sfGFP2*p*PaF, Figure S4: Nucleotide sequence of o-tDNA, Table S1: Primers used in this article.
