# Supplementary material for: Efficient Incorporation of Unnatural Amino Acids into Proteins with a Robust Cell-Free System
Source: Methods Protoc. 2019 Feb 12;2(1):16. doi: 10.3390/mps2010016 (PMC6481062; doi:10.3390/mps2010016)
Supplement: Supplementary file 1 [file mps-02-00016-s001.pdf]

Supplementary Materials for

Efficient Incorporation of Unnatural Amino Acids into Proteins With a Robust Cell-Free System

Wei Gao [1,2], Ning Bu [1,*] and Yuan Lu [2,3,4,*]

[1] College of Life Science, Shenyang Normal University, Shenyang 100034, Liaoning, China; weigao1122@gmail.com (W.G.); buning@synu.edu.cn (N.B.)

[2] Department of Chemical Engineering, Tsinghua University, Beijing 100084, China; yuanlu@tsinghua.edu.cn (Y.L.)

[3] Institute of Biochemical Engineering, Department of Chemical Engineering, Tsinghua University, Beijing 100084, China; yuanlu@tsinghua.edu.cn (Y.L.)

[4] Key Lab of Industrial Biocatalysis, Ministry of Education, Department of Chemical Engineering, Tsinghua University, Beijing 100084, China; yuanlu@tsinghua.edu.cn (Y.L.)

* Correspondence: buning@synu.edu.cn (N.B.); yuanlu@tsinghua.edu.cn (Y.L.); Tel.: +86-10-62780127 (Y.L.)

This PDF file includes:

Figure S1: Nucleotide and amino acid sequences of *p*PaFRS
Figure S2: Nucleotide and amino acid sequences of original sfGFP
Figure S3: Nucleotide and amino acid sequences of mutational sfGFP2*p*PaF
Figure S4: Nucleotide sequence of o-tDNA
Table S1: Primers used in this article

Supporting Figure S1. Nucleotide and amino acid sequences of pPaFRS.

Nucleotide sequence of pPaFRS.

ATGCATCACCATCACCATCATGGCGGAGCCATGGACGAGTTCGAAATGATTAAACGCA
ACACCAGCGAAATTATCTCTGAAGAAGAGCTGCGCGAGGTGCTGAAGAAAGACGAG
AAGAGCGCGGCCATTGGCTTTGAGCCGTCCGGTAAAATTCACCTGGGTCACTACCTGC
AAATCAAGAAGATGATTGATCTGCAAAACGCTGGTTTTGACATCATTATCCTGCTGGC
GGACCTGCACGCCTACCTGAATCAAAAGGGCGAGCTGGATGAGATTCGCAAGATCGG
CGACTACAATAAGAAAGTCTTCGAAGCCATGGGTTTGAAGGCTAAATACGTCTACGGT
AGCCCTTTTCAGCTGGATAAGGATTACACGTTGAATGTGTACCGTCTGGCGCTGAAAA
CCACGCTGAAACGCGCCCGTCGTTCCATGGAGCTGATTGCGCGCGAGGATGAGAATC
CAAAAGTTGCTGAGGTTATTTACCCTATTATGCAAGTTAATGCCATTCACTACGCGGGT
GTTGATGTTGCCGTCGGTGGTATGGAGCAACGCAAAATTCACATGCTGGCACGTGAAC
TGCTGCCGAAAAAGGTTGTCTGTATTCATAATCCGGTCCTGACCGGCCTGGATGGCGA
GGGTAAAATGAGCAGCAGCAAGGGTAACTTTATTGCAGTTGACGATAGCCCGGAAGA
AATCCGTGCGAAGATCAAGAAAGCGTACTGCCCGGCAGGCGTGGTTGAGGGTAACCC
GATCATGGAAATCGCCAAGTATTTTCTGGAATACCCACTGACGATTAAGCGCCCGGAG
AAATTTGGCGGCGACCTGACCGTCAACAGCTACGAGGAGCTGGAAAGCTTGTTTAAG
AACAAAGAACTGCATCCGATGCGCCTGAAAAACGCCGTGGCGGAAGAGCTGATTAA
GATTCTGGAACCAATTCGCAAACGTCTGTAA

Amino acid sequence of *p*PaFRS.

MHHHHHHGGAMDEFEMIKRNTSEIISEEELREVLKKDEKSAAIGFEPSGKIHLGHYLQIKK
MIDLQNAGFDIIILLADLHAYLNQKGELDEIRKIGDYNKKVFEAMGLKAKYVYGSPFQLDK
DYTLNVYRLALKTTLKRARRSMELIAREDENPKVAEVIYPIMQVNAIHYAGVDVAVGGME
QRKIHMLARELLPKKVVCIHNPVLTGLDGEGKMSSSKGNFIAVDDSPEEIRAKIKKAYCPAG
VVEGNPIMEIAKYFLEYPLTIKRPEKFGGDLTVNSYEELESLFKNKELHPMRLKNAVAEELIKI
LEPIRKRL*

Figure S2: Nucleotide and amino acid sequences of original sfGFP. Initiator AUG codon was indicated in green; and pPaF-encoding stop UAG codon was in bold and red. In the amino acid sequence, initiator (M) residues were green and in bold and pPaF residue was are highlighted and shown in red and bold. All the nucleotide and amino acid sequences of 6 x His tag were shown in purple.

Nucleotide sequence of original sfGFP

ATGCGTAAAGGCGAAGAGCTGTTCACTGGTGTCGTCCCTATTCTGGTGGAACTGGATG
GTGATGTCAACGGTCATAAGTTTTCCGTGCGTGGCGAGGGTGAAGGTGACGCAACTA
ATGGTAAACTGACGCTGAAGTTCATCTGTACTACTGGTAAACTGCCGGTACCTTGGCC
GACTCTGGTAACGACGCTGACTTATGGTGTTCAGTGCTTTGCTCGTTATCCGGACCATA
TGAAGCAGCATGACTTCTTCAAGTCCGCCATGCCGGAAGGCTATGTGCAGGAACGCA
CGATTTCCTTTAAGGATGACGGCACGTACAAAACGCGTGCGGAAGTGAAATTTGAAG
GCGATACCCTGGTAAACCGCATTGAGCTGAAAGGCATTGACTTTAAAGAAGACGGCA
ATATCCTGGGCCATAAGCTGGAATACAATTTTAACAGCCACAATGTTTACATCACCGCC
GATAAACAAAAAAATGGCATTAAAGCGAATTTTAAAATTCGCCACAACGTGGAGGAT
GGCAGCGTGCAGCTGGCTGATCACTACCAGCAAAACACTCCAATCGGTGATGGTCCT
GTTCTGCTGCCAGACAATCACTATCTGAGCACGCAAAGCGTTCTGTCTAAAGATCCGA
ACGAGAAACGCGATCATATGGTTCTGCTGGAGTTCGTAACCGCAGCGGGCATCACGC
ATGGTATGGATGAACTGTACAAACATCACCATCACCATCATTAA

Amino acid sequence of original sfGFP

MRKGEELFTGVVPILVELDGDVNGHKFSVRGEGEGDATNGKLTLKFICTTGKLPVPWPTLV
TTLTYGVQCFARYPDHMKQHDFFKSAMPEGYVQERTISFKDDGTYKTRAEVKFEGDTLVN
RIELKGIDFKEDGNILGHKLEYNFNSHNVYITADKQKNGIKANFKIRHNVEDGSVQLADHY
QQNTPIGDGPVLLPDNHYLSTQSVLSKDPNEKRDHMVLLEFVTAAGITHGMDELYKHHH
HHH*

Figure S3: Nucleotide and amino acid sequences of mutational sfGFP2pPaF. Initiator AUG codon was indicated in green; and pPaF-encoding stop UAG codon was in bold and red. In the amino acid sequence, initiator (M) residues were green and in bold and pPaF residue was are highlighted and shown in red and bold. All the nucleotide and amino acid sequences of 6 x His tag were shown in purple.

Nucleotide sequence of mutational sfGFP2*p*PaF

ATG**TAG**AAAGGCGAAGAGCTGTTCACTGGTGTCGTCCCTATTCTGGTGGAACTGGATG
GTGATGTCAACGGTCATAAGTTTTCCGTGCGTGGCGAGGGTGAAGGTGACGCAACTA
ATGGTAAACTGACGCTGAAGTTCATCTGTACTACTGGTAAACTGCCGGTACCTTGGCC
GACTCTGGTAACGACGCTGACTTATGGTGTTCAGTGCTTTGCTCGTTATCCGGACCATA
TGAAGCAGCATGACTTCTTCAAGTCCGCCATGCCGGAAGGCTATGTGCAGGAACGCA
CGATTTCCTTTAAGGATGACGGCACGTACAAAACGCGTGCGGAAGTGAAATTTGAAG
GCGATACCCTGGTAAACCGCATTGAGCTGAAAGGCATTGACTTTAAAGAAGACGGCA
ATATCCTGGGCCATAAGCTGGAATACAATTTTAACAGCCACAATGTTTACATCACCGCC
GATAAACAAAAAAATGGCATTAAAGCGAATTTTAAAATTCGCCACAACGTGGAGGAT
GGCAGCGTGCAGCTGGCTGATCACTACCAGCAAAACACTCCAATCGGTGATGGTCCT
GTTCTGCTGCCAGACAATCACTATCTGAGCACGCAAAGCGTTCTGTCTAAAGATCCGA
ACGAGAAACGCGATCATATGGTTCTGCTGGAGTTCGTAACCGCAGCGGGCATCACGC
ATGGTATGGATGAACTGTACAAACATCACCATCACCATCATTAA

Amino acid sequence of mutational sfGFP2*p*PaF

M*p*PaFKGEELFTGVVPILVELDGDVNGHKFSVRGEGEGDATNGKLTLKFICTTGKLPVPWPT
LVTTLTYGVQCFARYPDHMKQHDFFKSAMPEGYVQERTISFKDDGTYKTRAEVKFEGDTLV
NRIELKGIDFKEDGNILGHKLEYNFNSHNVYITADKQKNGIKANFKIRHNVEDGSVQLAD
HYQQNTPIGDGPVLLPDNHYLSTQSVLSKDPNEKRDHMVLLEFVTAAGITHGMDELYKH
HHHHH*

Figure S4: Nucleotide sequence of o-tDNA and structure of o-tRNA. The o-tDNA Sequence was indicated in red and bold. T7 promoter sequence was highlighted with yellow.

Nucleotide sequence of o-tDNA

GCTTTTAGATCTTAATACGACTCACTATAGGGAGACCGGCTGATGAGTCCGTGAGGACGAAACGGTACCCGGTACCGTC**CCGGCGGTAGTTCAGCAGGGCAGAACGGCGGACTCTAAATCCGCATGGCAGGGGTTCAAATCCCCTCCGCCGGACCA**

Structure of o-tRNA. Simulate on line via ViennaRNA Web Services

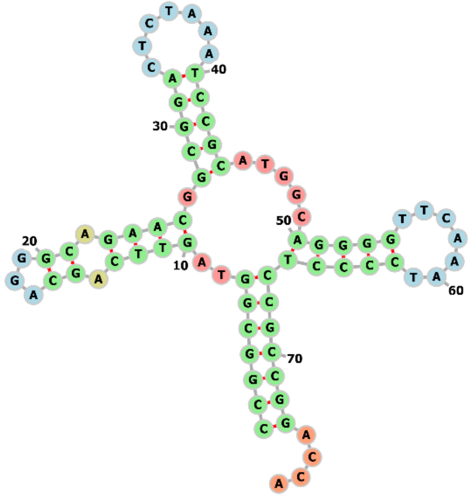

Table S1. Primers in this article.

Primers	Sequence
P1f	TTTCAGCTGGATAAGGATTACACGTTGAATGTGTACCGTCTGG
P1r	ACCACCGACGGCAACATCAACACCCGCGTAGTGAATGGCATTAAC
P2f	AGAAAGACGAGAAGAGCGCGGCCATTGGCTTTGAGCCGTC
P2r	TAATCCTTATCCAGCTGAAAAGGGCTACCGTAGACGTATTTAGCC
P3f	TTGATGTTGCCGTCGGTGGTATGGAGCAACGCAAAATTCACATG
P3r	CGCGCTCTTCTCGTCTTTCTTCAGCACCTCGCGCAG
P4f	TGGAGCCACCCGCAGTTCGAAAAGTAAGTCGACAAGCTTGCGGCCGC
P4r	TTACTTTTCGAACTGCGGGTGGCTCCATTTGTACAGTTCATCCATACCATG CGTGATGCCC
P5f	CATATGTAGAAAGGCGAAGAGCTGTTC
P5r	GCCTTTCTACATATGTATATCTCCTTCTTAAAGTTAAAC
P6f	CCCTCCGCCGGACCAAAGCTTGCGGCCGCACTC
P6r	TTAAGATCTAAAAGCGTCGACGGAGCTCGAATTCGGATCC
P7f	GCTTTTAGATCTTAATACGACTCACTATAGGGAGACCG
P7r	TGGTCCGGCGGAGGGGAT
